# Analysis of the Effects of Day-Time vs. Night-Time Surgery on Renal Transplant Patient Outcomes

**DOI:** 10.3390/jcm8071051

**Published:** 2019-07-18

**Authors:** Nesrin Sugünes, Anna Bichmann, Nadine Biernath, Robert Peters, Klemens Budde, Lutz Liefeldt, Thorsten Schlomm, Frank Friedersdorff

**Affiliations:** 1 Department of Urology, Charité-Universitätsmedizin Berlin, Corporate Member of Freie Universität Berlin, Humboldt-Universität zu Berlin, and Berlin Institute of Health, Charitéplatz 1, 10117 Berlin, Germany; 2 Department of Anesthesiology and Operative Intensive Care Medicine, Charité-Universitätsmedizin Berlin, Corporate Member of Freie Universität Berlin, Humboldt-Universität zu Berlin, and Berlin Institute of Health, Charitéplatz 1, 10117 Berlin, Germany; 3 Department of Nephrology, Charité-Universitätsmedizin Berlin, Corporate Member of Freie Universität Berlin, Humboldt-Universität zu Berlin, and Berlin Institute of Health, 10117 Berlin, Germany

**Keywords:** night-time renal transplantation, graft survival, patient survival/outcome, surgical complications

## Abstract

Sleep deprivation and disruption of the circadian rhythms could impair individual surgical performance and decision making. For this purpose, this study identified potential confounding factors on surgical renal transplant patient outcomes during day and night. Our retrospective cohort study of 215 adult renal cadaver transplant recipients, of which 132 recipients were allocated in the “day-time” group and 83 recipients in the “night-time” group, primarily stratified the patients into two cohorts, depending on the start time. Within a 24 h operational system, “day-time” was considered as being from 8 a.m. to 8 p.m. and “night-time” from 8 p.m. to 8 a.m.. Primary outcomes examined patient and graft survival after three months and one year. Secondary outcomes included the presence of acute rejection (AR) and delayed graft function (DGF), as well as the rate of postoperative complications. In log-rank testing, “day-time” surgery was associated with a significantly higher risk of patient death (*p* = 0.003), whereas long-term graft survival was unaffected by the operative time of day. The mean cold ischemia time (CIT), which was 12.4 ± 5.3 h in the “night-time” group, was significantly longer compared to 10.7 ± 3.6 for those during the day (*p* = 0.01). We observed that “night-time” kidney recipients experienced more wound complications. From our single-centre data, we conclude that night-time kidney transplantation does not increase the risk of adverse events or predispose the patient to a worse outcome. Nevertheless, further research is required to explore the effect of fatigue on nocturnal surgical performance.

## 1. Introduction

Kidney transplant outcomes have improved in recent years through novel technical approaches and immunosuppressive therapy [1,2,3,4]. There is still a deleterious impact of surgical complications on graft and patient survival [5,6]. Several risk factors of surgical complications have been identified, including donor and recipient characteristics, organ recovery and surgical implantation techniques [7]. Recipients with a prolonged cold ischemia time (CIT) have a greater risk for delayed graft function (DGF) and diminished long-term allograft survival [8]. To reduce CIT, surgery is initiated at any time of the day to preserve the organ quality. Further risk factors which are detrimental for patient outcome are human factors, including physical and mental fatigue and sleep deprivation, which are known to affect communication, attention and situational awareness, as well as psychomotor function [9,10]. It has been hypothesized that sleep deprivation reduces the performance of surgeons by affecting cognitive and fine motor skills [11,12]. In a technically demanding field, such as renal transplantation, meticulous preparation and excellent suturing techniques are required to prevent vascular and urologic complications [7]. The impact of physician fatigue on the medical error rate and clinical outcomes has been actively researched [13,14,15]. A number of studies demonstrated that operative outcomes were not related to sleep deprivation [16,17,18], whereas others link mental fatigue to surgical complication rates after general procedures [19]; and mortality after liver transplants [20]. To our knowledge, the literature regarding the impact of night-time surgery on outcomes after kidney transplantation is underrepresented and recent studies have reported conflicting results [21,22,23,24]. For this purpose, we conducted a retrospective cohort study to examine the association between the time of day of transplantation surgery (night-time vs. day-time) on surgical renal transplant patient outcomes. The primary outcomes examined were patient and graft survival after three months and one year. Secondary outcomes included the presence of acute rejection (AR) and DGF and other postoperative complications. We hypothesize, that renal transplantation surgery performed during the night-time would have inferior outcomes compared to those performed during the day.

## 2. Methods

We performed a retrospective cohort study of all adult patients undergoing cadaver renal transplantation at Charité University Hospital Campus Mitte, between 01.01.2011 and 31.12.14. Data on kidney transplantation and operative variables, as well as follow-up data, were obtained retrospectively from internal SAP (System, Anwendung, Produkte) and national TBase (Kidney Transplant Information System) electronic databases. The entire analysis was in adherence with correct scientific research work terms of the Charité Medical University of Berlin including full anonymization of patient data (‘Good Scientific Practice’, version 29/03/18).

### 2.1. Study Population

Transplants were stratified by the operative time of day. “Day-time” surgery was defined as surgery that started between 8:00 a.m. and 8:00 p.m. and “night-time” surgery was defined as surgery that started between 8:00 p.m. and 8:00 a.m. Twelve surgeons performed all the transplantations using standard surgical techniques. Kidneys were placed either in the right or left iliac fossa via an extraperitoneal approach. The renal graft vessels were anastomosed end-to-side to the recipient external or common iliac vessels. In all cases, except for one patient with urinary diversions (ileal conduit), a standard Lich–Gregoir ureteroneocystostomy was performed. A double-J ureteral stent was systematically inserted and removed six weeks later, followed by a urethral catheter for ten days postoperatively. All recipients received intravenous prophylactic antibiotics at the time of transplant. Graft function was monitored by Doppler ultrasound scanning, serum creatinine level and urine output measurements. The routine immunosuppression protocol that was initiated consisted of a triple regimen, including calcineurin inhibitors or a mammalian target of rapamycin inhibitors, mycophenolate mofetil (MMF), and steroids.

### 2.2. Data Collection

Patient and donor demographics and clinical data were collected by chart review. The parameters evaluated in this study were, recipient characteristics of age, gender, body mass index (BMI), comorbidities (hypertension, diabetes mellitus, cardiovascular disease, stroke and peripheral vascular disease), previous abdominal surgery, causation of end-stage renal disease, previous transplantation, duration of pre-transplant dialysis, and human leukocyte antigen (HLA) mismatches. The donor features were age, gender, BMI, site of donor kidney, number of graft arteries and the presence of graft vessels atherosclerosis. Perioperative factors included the surgeon’s experience (consultant, resident), cold and warm ischemia time (WIT), and incidence of intraoperative complications. CIT was defined as the time between the start of cold perfusion and removal of the renal allograft from ice. Warm ischemia time was defined as the time between the placement of the renal allograft into the iliac fossa of the recipient until revascularization of the kidney occurred.

### 2.3. Outcome Measures

The primary outcomes examined were patient and graft survival after three months and one year, respectively. Secondary outcomes included the presence of AR and DGF, as well as the rate of postoperative complications. Postoperative complications were examined for the first three months after surgery and defined according to the Clavien–Dindo classification system [20].

### 2.4. Statistical Analysis

Univariate comparisons were performed using the Chi-Square test or Fisher exact test for categorical variables. Continuous variables were tested with the non-paired Student t-test and the Mann–Whitney-U test for data with non-normal distribution. Categorical variables were displayed as n (%) and continuous variables mean ± standard deviation (SD); and nonparametric distribution as median (minimum-maximum). Patient and allograft survival rates were estimated using the Kaplan–Meier method and comparisons of survival rates were performed using the log-rank test. For all statistical measures, a *p*-value below 0.05 was considered statistically significant. Statistical analyses were performed using SPSS software (SPSS Inc., version 25, Armonk, NY, USA).

## 3. Results

The baseline characteristics and operative parameters in the two groups stratified according to the time surgery was performed are presented in Table 1. The two groups were similar with respect to most of the baseline characteristics, except for the higher distribution of male donors for the “day-time” group (*p* = 0.05). The mean CIT was 12.4 ± 5.3 h in the “night-time” group compared with 10.7 ± 3.6 for the “day-time” cohort (*p* = 0.01). The total operative time from skin incision to wound closure was similar in kidney transplants performed at all times. Considering the surgical expertise, 76.5% of “day-time” procedures were performed by a consultant compared to 72.3% during the “night-time” (*p* = 0.49). A total of six intraoperative surgical complications occurred in the overall cohort of 215 recipients (2.8%): renal artery stenosis (*n* = 2), renal vein injury (*n* = 2), renal vein thrombosis (*n* = 1) and iatrogenic bladder perforation (*n* = 1), which were immediately treated. The difference in incidence of intraoperative surgical complication was statistically insignificant with 3.8% (*n* = 5) during the day and 1.2% (*n* = 1) during the night (*p* = 0.34). We observed a higher incidence of DGF nocturnal operations with 54.2% compared to 47.7% in the “day-time” group (*p* = 0.35). The incidence of AR was 25% for “night-time” compared to 22% for “day-time” allograft recipients (*p* = 0.57). Table 2 shows patient outcomes.

### 3.1. Patient and Graft Survival

**Figure 1:**
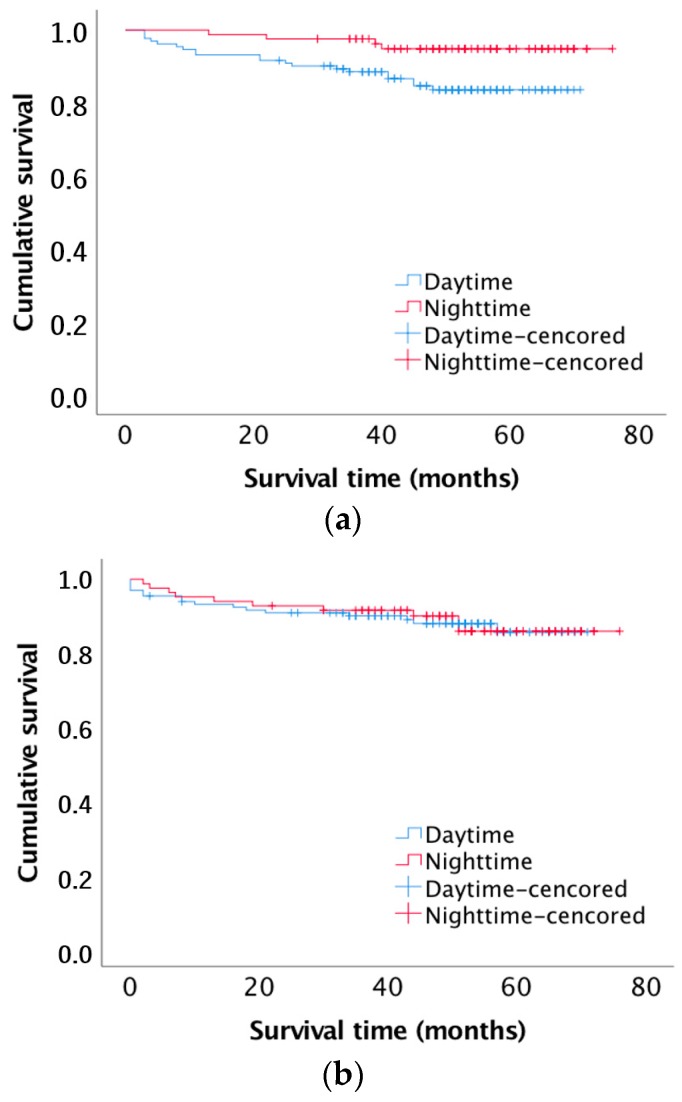
(**a**) Kaplan–Meier survival curve for patient survival after “day-time” and “night-time” renal transplantation (log-rank test 5.65; *p* = 0.017); (**b**) Kaplan–Meier survival curve for death censored graft survival of patients after “day-time” and “night-time” renal transplantation (log-rank test 0.014; *p* = 0.907).

The Kaplan–Meier survival curves for patient survival (Figure 1a) and death-censored allograft survival (Figure 1a) by status are shown in Figure 1. In log-rank testing, “day-time” operation was associated with a significantly higher risk of patient death (log-rank test 5.65; *p* = 0.017). During the first 12 months after surgery, a total of nine deaths occurred in the overall sample of 215 kidney transplants recipients (4.18%). No death occurred in the “night-time” kidney group within one year of transplantation, whereas two of the 132 “day-time” renal recipients died with a functioning transplant (one case of coronary heart disease, and one of malignancy) and seven patients died after returning to dialysis (all cases due to bacterial sepsis). Kaplan–Meier analyses demonstrated no statistically significant differences for death-censored graft survival (Figure 1b) between the “night-time” and “day-time” recipient cohorts (log-rank test 0.014; *p* = 0.907).

### 3.2. Early Graft Failure

During the first three months post-operation, graft failure was noticed in seven out of 132 “day-time” allograft recipients (5.3%), and in two out of 83 (2.4%) “night-time” transplant recipients (*p* = 0.49). In the “day-time” cohort, the most common cause of graft failure was primary non-function (*n* = 3), whereas recurrent disease, sepsis and death with functioning graft were noticed in the other cases, respectively. One “day-time” renal transplant recipient suffered an invasive fungal infection, which produced an allograft vessels aneurysm leading to graft loss. From the “night-time” group, two recipients (2.4%) lost the graft during the first three months after transplantation, due to AR and graft infection.

### 3.3. Postoperative Complications

One or more postoperative complications occurred in 74 out of the 132 “day-time” renal transplant recipients (56%) compared to 41 of the 83 “night-time” allograft recipients (49%) during the first three months post-operation (*p* = 0.34). Each category of complication assessed by the Clavien–Dindo grading system was analysed separately against the two-time groups, of which no category was significantly different (Table 3). In particular, “night-time” and “day-time” renal recipients did not differ significantly in the incidence of postoperative complications requiring medical or surgical reintervention (Clavien–Dindo Grade IIIa/b). Table 4 shows the number of operations that were performed within each time period and the incidence of surgical complications. The most common surgical complications in both groups included haemorrhagic events requiring blood transfusions or surgical intervention (17.2%), lymphoceles (10.7%), seromas (9.7%), and wound dehiscence (7%). A statistically insignificant higher incidence of wound complications among “night-time” kidney recipients was observed. The incidence of urologic complications was higher for the “day-time” surgery, which was also statistically insignificant. Among the 12 patients with urological complications, nine (6.8%) occurred within the “day-time” group and three (3.6%) during the “night-time” group. Five patients (2.3%) were treated with interventional procedures and two (0.7%) received surgical intervention under general anaesthesia. Ureteric necrosis occurred in one “day-time” renal recipient, which was treated with ureteric re-implantation. The incidence of vascular complications within three months post-transplantation was, respectively, 4.5% for “day-time” and 2.4% for “night-time” surgery. In four cases (1.9%), an early secondary surgical intervention was required for vascular complications. Renal artery stenosis occurred in 0.9% of all recipients. Renal artery aneurysm and renal vein thrombosis occurred equally at the rate of 0.5%.

## 4. Discussion

Over the past decade, increased understanding of the effects of shift work and sleep deprivation on neurocognitive functions and physicians health has been established [25]. A single-center study by Rothschild et al. suggested that surgical outcomes were compromised if surgeons had less than six hours of sleep per shift [19]. Traffinder et al. reported that fatigued surgeons made 20% more errors and took 14% longer to perform laparoscopic tasks [26]. On the other hand, studies have demonstrated that outcomes of surgical procedures may not be adversely affected by fatigue or disruption of the normal circadian rhythm [16,17,18]. Five studies with limited numbers of transplants have previously assessed this issue by focusing on the impact of night-time surgery on graft outcome or complications in patients undergoing renal transplantation [21,22,23,24]. Only one single-center study, performed by Fechner et al., demonstrated that night-time surgery carries a higher risk of adverse events and poorer outcomes, particularly driven by higher rates of vascular complications [21]. Kienzel et al. reported that, if transplantations were postponed until the next morning, the increase in CIT would decrease the long-term survival [22]. Seow et al. did not observe an adverse effect of night-time surgery on patient outcomes but highlighted surgical clinical expertise to be a crucial factor for surgical complications [23]. Several limitations need to be considered in the interpretation of the contradictory results. Most studies published to date reported great variability in the methodology and outcome measures. In addition, the definition and understanding of sleep deprivation varied widely among previous investigators. Mentioned studies are frequently single-center and reported the results of a small groups of surgeons, which limits the generalizability. In the present study, we did not find any significant impact of night-time kidney transplant surgery on outcomes including three-month and one-year patient or allograft survival, postoperative complications, DGF or AR in the first year. Our analysis revealed a variable incidence of complications among the different time groups and we could not determine any consistent trend. While the incidence of vascular, haemorrhagic and urological complications was greatest in the “day-time” operative group, wound complications occurred more often among recipients of “night-time” transplants without statistical significance. The mean CIT was slightly longer among those who underwent night-time transplant operations compared to the “day-time” cohort. We observed diminished patient survival among “day-time” renal transplant recipients compared to “night-time” allograft recipients, whereas long-term graft survival was unaffected by the time of day. With no significant difference in baseline characteristics, except for the slighter higher distribution of male donors in the “day-time” cohort, the reasons for this observation are still unclear. We have controlled for a majority of the clinically meaningful variables available to us in this data set, but it is possible that yet unidentified biologic factors could account for the difference in patient survival between the “night-time” and “day-time” cohorts. With this in mind, there is an urgent need for research in order to clarify the biological consequences of sleep disturbance and fatigue among renal transplant patients. The influence of circadian rhythmicity on physiologic functions related to renal cells, including blood pressure control and homeostasis regulation, is a well-studied phenomenon [27,28,29,30]. Evidence suggests that reduced sleep duration and disturbed circadian rhythms may increase sympathetic nervous system stimulation, increase blood pressure, and impair metabolic regulation [31,32,33]. Thus, misalignment of intrinsic circadian rhythms with environmental time may contribute to poor kidney functioning and renal injury among kidney transplant recipients and donors. Future study is required to clarify this issue. There may be several possible explanations for the lack of ‘night-time effect’ on outcomes after renal transplantation in this study. Recent studies have demonstrated that there is inter-individual variability in vulnerability to cognitive deficits from sleep loss and the ability to sustain effective neurocognitive performance [34,35], suggesting a reason why there were no differences between the “day-time” and “night-time” cohorts in our study. Van Dongen et al. reported differences in endogenous regulatory processes among individuals, which may affect their tolerance for shift work and cognitive performance during work shifts [36]. Performance adaption across successive shifts has been observed [37,38]. Leff et al. suggested improvement in technical procedural skills across remaining night shifts may be due to ongoing learning or adaption to chronic fatigue [37]. When considering the impact of nocturnal shift work on surgical performance, it is essential to also consider the effects of societal and environmental forces that may contribute to the biological consequences of circadian misalignment. It is known, that there is a detrimental effect of noise inside the operating room on the performance of surgeons and anaesthesiologists [39]. The exposure to excessive operating room noise and distractions during the main day-time business hours may impair cognitive skills. Other factors influencing the performance of a surgeon, such as leadership and communication may be at least as important as technical skills and the number of hours slept [40]. In addition, the use of caffeine and periods of short naps may mitigate the potential risks associated with sleep deprivation [41]. A study of this nature has some limitations, primarily through its retrospective design. The small overall number of patients and individual complications in our cohort might weaken the conclusions of our pilot-study and limits the power to detect differences. To assess severity, we additionally categorised all postoperative complications using the Clavien–Dindo classification system. Although this system has been proven to be reproducible and applicable with minimal interobserver variability, it has some limitations [42]. Data regarding a surgeon’s subjective perception of fatigue, resting time and quantification of sleep deprivation were not available and could not be included in the analysis. It is further possible, that transplant surgeons perform day-time procedures beginning at 8 am after being ‘on-call’ overnight. With that in mind, one may argue whether the classification based on time group selections assumes that day-time surgeons are well rested, and perform better than night-time surgeons regardless of their overall workload. We cannot lose sight of other potential variables such as the effect of procurement-related organ lesions on renal transplant outcome. Data concerning surgeons’ fitness before procurement were not available. Further investigation is needed aiming to record errors during organ procurement related to surgeons’ fatigue.

## 5. Concluding Remarks and Future Directions

To date, there are very few reports on the effect of night-time surgery on renal transplant outcomes. We, therefore, believe that the initial results from this pilot-study are a welcome addition to the urological literature and provide encouragement for further analysis. We concluded that night- time kidney surgery does not carry a higher risk of adverse events and poorer outcome among patients undergoing renal transplantation. Consequently, kidney transplantation should be immediately performed regardless of the time of the day, with the known adverse effects of prolonged CIT. However, in order to fully assess the effects of sleep deprivation and circadian rhythm disturbance on surgical performance in kidney transplantation, prospective research involving larger cohorts is needed. Therefore, among other things, a transparent evidence-based assessment of the level of fatigue, shift intensity and sleep quality in medicine, especially in the field of surgery, is required. Moreover, systems-based interventions, as well as individual coping strategies and experiences that mitigate the effects of fatigue and disruption of the circadian rhythms, should be taken into consideration. In addition, there is a need for future research focusing on the impact of sleep displacement and circadian misalignment on renal functioning among recipients and donors in the field of kidney transplantation.

## Figures and Tables

**Table 1:** Recipient and donor characteristics and operative details. Results are presented as mean and standard deviations or as absolute and relative frequencies; h—hours; min—minutes; ESRD—end-stage renal disease; * statistically significant.

Donor Characteristics	All (*n* = 215)	8:00 a.m.–8:00 p.m. (*n* = 132)	8:00 p.m.–8:00 a.m. (*n* = 83)	*p*-Value
Age (years)	54.2 ± 14.8	55.2 ± 15.1	52.5 ± 14.3	0.19
Male gender	114 (53.0%)	77 (58.3%)	37 (44.6%)	0.05
BMI (kg/m^2^)	25.8 ± 4.4	25.8 ± 4.2	25.8 ± 4.8	0.88
Right kidney side	107 (49.8%)	66 (50.0%)	41 (49.4%)	0.93
Multiple renal arteries (%)	45 (20.9%)	28 (21.2%)	17 (20.5%)	0.90
Atherosclerosis of graft vessels	128 (59.5%)	77 (58.3%)	51 (61.4%)	0.65
**Recipient Characteristics**				
Age (years)	53.3 ± 14.7	54.6 ± 14.6	51.1 ±14.7	0.12
Age > 65 years	64 (29.8%)	44 (33.3%)	20 (24.1%)	0.15
Male gender	120 (55.8%)	78 (59.1%)	42 (50.6%)	0.22
BMI (kg/m^2^)	25.8 ± 4.4	26.2 ± 4.4	25.2 ± 4.5	0.12
**Cause of ESRD**				
Glomerulonephritis	85 (39.5%)	52 (39.4%)	33 (39.8%)	
Hypertension/renovascular	41 (19.1%)	25 (18.9%)	16 (19.3%)	
Polycystic kidney disease	31 (14.4%)	20 (15.2%)	11 (13.3%)	
Diabetes mellitus	16 (7.4%)	10 (7.6%)	6 (7.2%)	
Interstitial nephritis	8 (3.7%)	5 (3.8%)	3 (3.6%)	
System diseases	8 (3.7%)	4 (3.0%)	4 (4.8%)	
Reflux nephropathy	6 (2.8%)	3 (2.3%)	3 (3.6%)	
Congenital uropathy	4 (1.9%)	2 (1.5%)	2 (2.4%)	
Other	12 (5.6%)	8 (6.1%)	4 (4.8%)	
Unknown	4 (1.9%)	3 (2.3%)	1 (1.2%)	
Re-transplantation	29 (13.5%)	17 (12.9%)	12 (14.5%)	0.74
Duration on dialysis (days)	2304 ± 1155	2331 ± 1145.5	2261 ± 1174.6	0.67
Mean HLA-mismatches	2.5 ± 1.5	2.5 ± 1.5	2.5 ± 1.5	0.95
**Co-Morbidities**				
Diabetes mellitus	44 (20.5%)	29 (22.0%)	15 (18.1%)	0.49
Hypertension	184 (85.6%)	110 (83.3%)	74 (89.2%)	0.24 *
Pre-transplant cardiovascular disease	47 (21.9%)	33 (25.0%)	14 (16.9%)	0.16
Stroke	18 (8.4%)	11 (8.3%)	7 (8.4%)	0.98
Peripheral vascular disease	21 (9.8%)	7 (5.3%)	14 (16.9%)	0.05
Pre-transplant abdominal surgery	84 (39.1%)	56 (42.4%)	28 (33.7%)	0.20
**Operation Characteristics**				
Total operative time (min)	203 ± 46.3	203.5 ± 44.4	202.3 ± 49.6	0.85
Warm ischemia time (min)	51.2 ± 12.3	51.4 ± 12.1	50.8 ± 12.6	0.74
Cold ischemia time (h)	11.4 ± 4.5	10.7 ± 3.6	12.4 ± 5.3	0.01 *
Consultant	161 (74.9%)	101 (76.5%)	60 (72.3%)	0.49
Intraoperative complication	6 (2.8%)	5 (3.8%)	1 (1.2%)	0.34

**Table 2:** Graft and recipient outcome, * statistically significant.

	All (*n* = 215)	8:00 a.m.–8:00 p.m. (*n* = 132)	8:00 p.m.–8:00 a.m. (*n* = 83)	*p*-Value
Overall patient survival				0.017 *
At 3 months	212 (98.6%)	129 (97.7%)	83 (100%)	
At 1 year	206 (95.8%)	123 (93.2%)	83 (100%)	
Death censored graft survival				0.907
At 3 months	207 (96.3%)	126 (95.5%)	81 (97.6%)	
At 1 year	202 (93.9%)	123 (93.2%)	79 (95.2%)	
Delayed graft function	108 (50.2%)	63 (47.7%)	45 (54.2%)	0.350
Acute rejection rate	50 (23.3%)	29 (22%)	21 (25.3%)	0.570
**Serum creatinine (mg/dL) after transplantation median (range)**				
1 week (*n* = 213)	4.4 (0.93–14.51)	4.4 (0.93–12.99)	4.4 (1.06–14.51)	0.730
4 weeks (*n* = 212)	1.72 (0.68–17.0)	1.74 (0.68–17.0)	1.71 (0.80–7.16)	0.710
24 weeks (*n* = 205)	1.45 (0.59–4.42)	1.45 (0.59–4.42)	1.45 (0.71–3.35)	0.660
60 weeks (*n* = 200)	1.38 (0.46–4.71)	1.39 (0.46–4.71)	1.34 (0.67–2.49)	0.270
Follow-up (months)	49.2 ± 14.6	47.1 ± 15.6	52.50 ± 12.4	0.008 *

**Table 3:** Postoperative complications with Clavien–Dindo Classification. Results are presented as absolute and relative frequencies. * If more than one occurred per case, according to patient records, the complication with the highest degree was selected (Minor I+II, Major complications IIIa-IVb, Mortality V).

	All (*n* = 215)	8:00 a.m.–8:00 p.m. (*n* = 132)	8:00 p.m.–8:00 a.m. (*n* = 83)	*p*-Value
**Complications (all grades)**	115 (46.5%)	58 (43.9%)	42 (50.6%)	0.340
Grade of complication *				
I	25 (11.6%)	17 (12.9%)	8 (9.6%)	0.470
II	39 (18.1%)	24 (18.2%)	15 (18.1%)	0.984
IIIa	15 (7.0%)	11 (8.3%)	4 (4.8%)	0.325
IIIb	28 (13.0%)	16 (12.0%)	12 (14.5%)	0.620
IVa	3 (1.4%)	3 (2.3%)	0 (0%)	0.286
IVb	4 1.9%)	2 (1.5%)	2 (2.4%)	0.640
V	1 (0.5%)	1 (0.8%)	0 (0%)	1.000

**Table 4:** Incidence of surgical complications. Incidence is expressed as percentages (%) of total number (*n*) of patients.

Surgical Complications	All (*n* = 215)	8:00 a.m.–8:00 p.m. (*n* = 132)	8:00 p.m.–8:00 a.m. (*n* = 83)	*p*-Value
**Vascular**				
Renal artery stenosis	2 (0.9%)	1 (0.8%)	1 (1.2%)	1.0
Renal vein thrombosis	1 (0.5%)	1 (0.8%)	0	1.0
Iliac artery thrombosis	1 (0.5%)	1 (0.8%)	0	1.0
Renal artery aneurysm	1 (0.5%)	1 (0.8%)	0	1.0
Renal anastomotic leak	1 (0.5%)	0	1 (1.2%)	0.39
Renal pole infarct	1 (0.5%)	1 (0.8%)	0	1.0
Coeliac Trunk stenosis	1 (0.5%)	1 (0.8%)	0	1.0
**Haemorrhagic**				
Haematoma	31 (14%)	20 (15.2%)	11 (13.3%)	0.70
Haemorrhage	6 (2.8%)	3 (2.3%)	3 (3.6%)	0.56
**Urological**				
Urinary leak	3 (1.4%)	2 (1.5%)	1 (1.2%)	1.0
Urethral necrosis	1 (0.5%)	1 (0.8%)	0	1.0
Urethral stent complication	1 (0.5%)	1 (0.8%)	0	1.0
Urethral stricture	2 (0.9%)	1 (0.8%)	1 (1.2%)	1.0
Bladder outflow obstruction/Blood clot retention	4 (1.9%)	4 (3.0%)	0	0.16
**Wound related**				
Lymphocele	23 (10.7%)	13 (9.8%)	10 (12%)	0.53
Seroma	17 (7.9%)	9 (6.8%)	8 (9.6%)	0.46
Wound dehiscence	15 (7%)	9 (6.8%)	6 (7.3%)	0.89
Impaired wound healing	3 (1.4%)	0	3 (3.6%)	0.06
Wound infection	3 (1.4%)	3 (2.3%)	0	0.29
